# Volume Flow Measurements in Arteriovenous Dialysis Access in Patients with and without Steal Syndrome

**DOI:** 10.1155/2013/328601

**Published:** 2013-08-27

**Authors:** Charudatta S. Bavare, Jean Bismuth, Hosam F. El-Sayed, Tam T. Huynh, Eric K. Peden, Mark G. Davies, Alan B. Lumsden, Joseph J. Naoum

**Affiliations:** ^1^Department of Cardiovascular Surgery, The Methodist DeBakey Heart and Vascular Center, The Methodist Hospital, 6550 Fannin Street, Suite 1401 Houston, TX 77030, USA; ^2^Lebanese American University and University Medical Center Rizk Hospital, Beirut, Lebanon

## Abstract

*Introduction*. Dialysis associated steal syndrome (DASS) constitutes a serious risk for patients undergoing vascular access operations. We aim to assess the measured volume flow using ultrasound in patients with clinically suspected steal syndrome and determine differences in flow among types of arteriovenous (AV) access. *Methods*. Patients with permanent hemodialysis access with and without ischemic steal underwent duplex ultrasound (US) exams for the assessment of volume flow and quantitative evidence of hemodynamic steal. Volume flow was measured in the proximal feeding artery. *Results*. 118 patients underwent US of which 82 (69.5%) had clinical evidence of steal. Women were more likely to develop steal compared to men (chi-squared test *P* < 0.04). Mean volume flow in patients with steal was 1542 mL/min compared to 1087 mL/min (*P* < 0.002) in patients without evidence of steal. A significant difference in flow volumes in patients with and without steal was only seen in patients with a brachial-cephalic upper arm AV fistula (AVF) (*P* < 0.002). When comparing different types of access with steal, brachial-cephalic upper arm AVFs had higher volume flows than the upper extremity AV graft (AVG) group (*P* = 0.04). *Conclusion*. In patients with DASS, women were more likely to develop steal syndrome. Significantly higher volume flows were seen with brachial-cephalic upper arm AVF in patients with steal compared to those without. A physiologic basis of this US finding may be present, which warrants further study into the dynamics of flow and its relationship to the underlying peripheral arterial pathology in the development of ischemic steal.

## 1. Introduction

Creating and maintaining a functional hemodialysis access conduit is challenging. With an increasing number of patients needing hemodialysis per year, the demand for a durable access with minimum complications is also increasing. Among the postoperative complications, dialysis associated steal syndrome (DASS) is the most morbid, often resulting in significant neurologic injury or tissue loss. Clinical risk factors previously identified in patients at risk for development of DASS include age greater than 60 years, female gender, diabetes, previous limb procedures, and type of fistula constructed [1–5]. DASS is a relatively uncommon phenomenon, occurring in 1%–10% of cases [3]. There are no reliable methods of predicting its development, and management is challenging. Preservation of the existing access and relief of the ischemia are a priority in the treatment of DASS.

Bussell and associates described DASS in patients with a radial-cephalic arteriovenous fistula (AVF) by using pneumatic plethysmography [6]. The diagnosis is clinically suspected when there are new symptoms either immediately after access creation or subsequently on followup after maturation. It is confirmed clinically by physical exam demonstrating a cold distal extremity, pain, pallor, diminished, or absent peripheral pulses in the extremity, muscle wasting, sensory impairment, or even ulceration or gangrene in the late cases [7, 8]. A noninvasive hemodynamic assessment can be performed by measuring the distal and/or forearm Doppler pressure and by recording digital pulse wave plethysmography or by comparing digital pressure measurements with and without manual compression of the arteriovenous (AV) access [9, 10]. Accompanying volume flows in the feeding artery have been previously measured but have not consistently shown to correlate to the presence or absence of steal [11]. We proposed that there might be a correlation between volume flows and presence of steal in AV access and that there may be differences between the types of access. 

## 2. Materials and Methods

### 2.1. Study Design

This was a retrospective review of 118 consecutive patients who underwent US exams of their functioning hemodialysis access during a 30-month period. Eighty-two of those patients had evidence by clinical and physical examination of having steal syndrome. Collected data included gender, type of AV access, and volume flow measurements in the proximal feeding artery. 

### 2.2. Study Setting

Academic Medical Center with 1000 beds in a catchment area of 5 million people. It is a tertiary and quaternary referral facility. During the study period, a total of 1187 AV access cases were performed. 

### 2.3. Technique

Duplex examinations were performed based on a standardized protocol using a Philips iU22 xMatrix color duplex scanners (Phillips Healthcare, Andover, MA) and a L 7–4 MHz linear transducer with patients in a recumbent position. Transverse and longitudinal B-mode and color flow images were obtained along at least 10 cm or more of the arterial inflow and the arterial anastomosis [11]. Waveforms were recorded from a small sampling volume placed in the central flow stream at attempted angles of 60° relative to vessel walls of the feeding artery. Velocity sampling was done at multiple sites proximal and distal to the anastomosis and the highest volume flow rate selected [11, 12]. A marked or significant reversal of flow especially in the brachial artery distal to the anastomosis was suggestive of severe steal and consequent distal ischemia as described by Zamani and colleagues [7] and suggested by van Hoek and associates [13]. Plethysmography probe measurements before and after access compression were used to document doubling of maximum wave amplitude upon compression of the fistula outflow [2, 9, 10]. 

### 2.4. Statistics

Student's *t*-*test* was used to compare continuous variables (volume flows). The chi-squared test was used to compare categorical variables (men and women with and without steal syndrome). 

## 3. Results

One hundred and eighteen consecutive patients of which 82 (69.5%) had clinical evidence of steal were evaluated by duplex ultrasound (US).  Table 1 details the number of men and women presenting with and without steal syndrome. Women were more likely to present with steal than men (chi-squared test *P* < 0.04).

 Mean volume flow in patients with steal was 1542 mL/min compared to 1087 mL/min (*P* < 0.002) in patients without evidence of steal. However, a significant difference in flow volumes between those with steal and without steal syndrome was only seen in patients with brachial-cephalic upper arm AVF (*P* < 0.002). When comparing between types of access in patients with steal syndrome, brachial-cephalic upper arm AVFs had significantly higher volume flows than the upper extremity AV graft (AVG) group (*P* = 0.04).  Table 2 shows the mean volume flow by access type in patients with and without steal.

## 4. Discussion

Complications of vascular access, including thrombosis, bleeding, infection, pseudoaneurysm, and distal ischemia [14, 15] are a large cause of morbidity in the hemodialysis population in the United States [16]. Though uncommon among these, the most morbid is hand ischemia or steal syndrome. It can often result in significant neurologic injury, motor deficit, or tissue loss. Management has proven to be a challenge partly because of the desire to maintain access while alleviating the ischemia in this difficult population with often advanced peripheral vascular disease.

Steal phenomenon is particularly frequent in patients with forearm and upper arm AVFs and in patients with prosthetic straight or loop grafts [15]. Because of low resistance in the venous outflow, the AV access takes not only the antegrade flow into the feeding artery but also “steals” retrograde flow from the hand via the palmar arch and jeopardizes its adequate perfusion. Interestingly, reversed blood flow in the artery distal to the anastomosis has been observed in radial-cephalic AVFs [6] but has not been documented in brachial artery based AV access [13]. Some minimal element of “steal” may occur in 75%–90% of patients after creation of the vascular access [15, 16]. Usually the steal phenomenon is clinically silent, and the patient remains asymptomatic. The steal phenomenon is converted into a steal syndrome when compensatory mechanisms to maintain peripheral arterial perfusion fail. The steal syndrome is characterized by pain at rest, pain during hemodialysis sessions, ulcerations, mostly acral necrosis, and even tissue loss. Preoperative risk factors for a steal syndrome are female gender [10], age > 60 years, and diabetes mellitus [1] construction of an autogenous fistula, multiple previous operations on the limb, and use of the brachial artery as the donor vessel [1–5]. Our series corroborates these findings with more steal phenomenon seen in women and in patients with brachial-cephalic upper arm AVFs. 

Although angiography has been considered as the gold standard for imaging of vascular access abnormalities, duplex ultrasound may be helpful in some aspects since it provides information both on the morphology and on the function of the vascular access. In addition, US offers the advantage of a noninvasive procedure with lower cost and avoidance of contrast media. Volume flow measurements traditionally have been used to diagnose fistula dysfunction with lower flow rates corresponding to higher failure rates [17, 18]. For instance, Bay and associates described a series of over 2700 patients in whom serial volume flow measurements were done to predict failure rates over followup [19]. 

Some authors have argued that dialysis access volume flow and the presence of steal are related [13]. Our series suggests a correlation between volume flow rates and steal syndrome. Overall we found a significantly higher flow rate in access with steal syndrome than in those without steal. In particular, a significant difference in flow volumes in patients with and without steal was only seen with a brachial-cephalic upper arm AVFs. This finding may be clinically relevant and may have potential implications for its surgical management. Owing that our data suggest that brachial-cephalic AVFs develop steal in part due to high volume flow rates compared to those without steal, treatment may be potentially influenced by the decision to decrease or reduce the flow through the AVF. Thus, flow reduction techniques such as a simple plication of the inflow may be considered in this instance [20]. This is based on the premise that increasing fistula resistance or decreasing the flow through it will indirectly increase perfusion to the distal extremity [21]. Tordoir and associates have suggested that blood shunting through the AVF may cause stealing of blood and hypoperfusion of distal tissues [22]. In addition, they have shown that high-flow AVFs have a greater risk of ischemia than AVFs with normal flow volumes, with the caveat that when combined with peripheral arteriosclerotic disease the latter may also lead to ischemia. They suggest that augmentation of arterial inflow by interventional techniques and/or AVF blood flow-reducing surgical procedures may eliminate pain and heal ulcers in this particular case. 

 We did not find an overall significant difference between flow volumes when comparing upper arm AV grafts, brachial-basilic upper arm transposition, or radial-cephalic forearm AVFs with and without steal. In previous work by van Hoek and colleagues, the intensity of steal was not related to the magnitude of access flow [13]. However, similar to our report, individuals with brachial-cephalic upper arm AVFs were at higher risk of developing complaints associated with reduced hand circulation compared to those with a radial-cephalic forearm AVF or an upper arm AVG. Fistulas, unlike grafts, have an intact endothelial lining that allows them to actively dilate and remodel over extended periods. In addition, fistulas have side branches that reduce resistance to flow and ligation of accessories or spontaneous occlusion of side branches within a fistula increases resistance and results in an access that hemodynamically mimics the profile of a graft [8]. In our series, none of the patients with AVFs had branch ligations. These factors may partially explain our finding of higher volume flows observed in patients with steal having a brachial-cephalic upper arm AVF compared to the AVG group. 

The present study has several limitations. First, the scope of this retrospective study focused on US evaluation of volume flows in AV dialysis access. We did not capture data on the status of the forearm and digital arteries nor measured digital pressures or indices. Second, we did not correlate the US findings with angiographic data. Thus, we do not have a picture of the underlying arterial pathology that clearly contributes to the multifactorial nature of steal. Similarly, by not correlating with arteriographic images, we do not have the ability to evaluate the formation of arterial collaterals as a potential compensatory mechanism in physiologic steal [21]. Third, we did not collect data on patient comorbidities or systemic hemodynamics. Access flow is related to the cardiac output and cardiac index. Thus, in patients with congestive heart failure or those with decrease heart function, volume flow measurements will be affected negatively. Similarly, increases in peripheral vascular resistance (PVR) as often seen in diabetics will also affect access flow. Work by Wijnen and colleagues describes this relationship and found that access flow was significantly and positively related to the cardiac output and cardiac index and inversely related to PVR [23]. We limited the investigation to US derived volume flow measurements and its correlation with the presence of ischemic steal syndrome. We did not explore the therapeutic interventions that these patients may have had. This has important implications precisely if we want to quantitate and compare the volume flow after intervention and assess its correlation with the persistence or absence of symptoms of steal and AV access function. Finally, we believe that our observations may contribute to the understanding of DASS, and we intend that the US data generated in this work be validated by prospective studies in the future. 

## 5. Conclusion

Dialysis associated steal syndrome is a seriously morbid complication of AV access creation. Accurate history taking, physical exam, and noninvasive US studies are important in confirming the diagnosis. In patients with DASS, women were more likely to develop steal syndrome. Significantly higher volume flows were seen with a brachial-cephalic upper arm AVF in patients with steal compared to those without. This may have potential implications in the management of this complication. A physiologic basis of this US finding may be present, which warrants further investigation into the dynamics of flow and resistance in different AV access conduits and their interplay with the underlying arterial pathology in the development of ischemic steal syndrome.

## Figures and Tables

**Table 1:** Patients presenting with and without steal syndrome.

Steal syndrome	Men (%)	Women (%)	Total (%)
Not present	17 (42.5)	19 (24.4)	36 (30.5)
Present	23 (57.5)	59 (75.6)*	82 (69.5)

Total	40 (34)	78 (66)	118 (100)

*Chi-squared test *P* < 0.04.

**Table 2:** Types of access with corresponding volume flows in patients with and without steal.

Type of access	*n*	% with steal	Mean volume flow in access without steal (mL/min)	Mean volume flow in access with steal (mL/min)
Radial-cephalic forearm	17	76.5	887	1247
Brachial-cephalic upper arm	49	73.4	1032*	1701*
Brachial-basilic upper arm transposition	29	65.5	1191	1535
AVG upper extremity	23	63.6	1149	1413

Total	118	69.5	1087*	1542*

*P < 0.002.
